# Evaluation and application of an innovative portable field endoscope: addressing battlefield and biosafety concerns

**DOI:** 10.1186/s40779-025-00644-w

**Published:** 2025-09-15

**Authors:** Gang Sun, Jia-Qi Zeng, Jun-Ling Wu, Shu-Fang Wang, Li-Hua Peng, Bin Yan, Fei Pan, Yi Li, Guan-Zhou Zhou, Xiao-Dong Chen, Zi-Kai Wang, Xiang-Dong Wang, Wan-Yuan Lian, Yun-Sheng Yang

**Affiliations:** 1 Department of Gastroenterology and Hepatology, the First Medical Center, Chinese PLA General Hospital, Beijing, 100853 China; 2 Department of Medical College of Chinese PLA, Beijing, 100853 China; 3 Department of Huizhou, Xzing Technology Co., Ltd., Huizhou, 516025 Guangdong China; 4 Department of Medical College, Nankai University, Tianjin, 300071 China; 5 College of Precision Instrument and Opto-Electronics Engineering, Tianjin University, Tianjin, 300072 China; 6 Department of National Clinical Research Center for Geriatric Diseases, Chinese PLA General Hospital, Beijing, 100853 China

**Keywords:** Portable gastrointestinal endoscopy, Gastrointestinal bleeding, Colonoscopy, Endoscopic procedures, Field endoscopy

## Abstract

**Background:**

At present, no commercially available endoscopic system is specifically designed for use in the battlefield, disaster relief, or unique environments with biosafety concerns. Therefore, this limitation stems from challenges such as limited portability, reliance on stable power, complex disinfection processes, and the risk of incomplete sterilization. To address these challenges, we developed a novel portable endoscopic system and evaluated its safety and effectiveness in both routine settings and specialized scenarios, including the global pandemic caused by a novel coronavirus, which represents an environment with biosafety concerns.

**Methods:**

After sample size calculation, 30 patients underwent esophagogastroduodenoscopy (EGD) or colonoscopy using the YunSendo (the experimental group) and Olympus systems (the control group) in a randomized order. Operation time, image quality, operational performance, lesion detection, and safety were assessed. Ten emergency patients with suspected upper gastrointestinal bleeding received bedside treatment using the YunSendo system during the global pandemic caused by a novel coronavirus. Clinical outcomes in emergency endoscopic treatment were assessed.

**Results:**

No significant differences were observed between the YunSendo and Olympus groups in terms of image quality, lesion detection, and overall procedural performance. YunSendo facilitated biopsy and colonic polyp removal; no adverse endoscopy events were reported. YunSendo successfully executed diagnostic and therapeutic procedures in emergencies, with no observed mortality at 1, 7, or 30 d, no rebleeding at 1 or 30 d, and no cross-infection rates.

**Conclusions:**

The performance of the YunSendo portable endoscopic system was comparable to the Olympus system in terms of key metrics, demonstrating its utility in urgent scenarios. This novel system is particularly promising for medical rescue and military missions and for addressing battlefield and biosafety concerns.

**Supplementary Information:**

The online version contains supplementary material available at 10.1186/s40779-025-00644-w.

## Background

Gastrointestinal (GI) endoscopy is essential for diagnosing and treating various GI disorders, its use and demand have grown significantly in China. According to the statistics, in 2019, 38.73 million endoscopic procedures were performed nationwide, with an increase of 34.62% compared to 2012 [1, 2]. This growth may be attributed to the development of endoscopic technology, which offers minimally invasive advantages for GI examination and treatment compared to traditional surgical methods [3].

Despite stringent cleaning protocols, the infection rate associated with GI endoscopy remains a significant concern. This is largely due to inadequate disinfection, which can lead to patient-to-patient transmission of pathogens, including multidrug-resistant *Pseudomonas*, *Salmonella, Helicobacter pylori, Klebsiella, Enterobacter*, and other intestinal bacteria, as well as parasites [4–7]. In response, military medicine emphasizes the importance of rigorous sterilization and infection control to reduce these risks and improve recovery outcomes in challenging environments [8]. During outbreaks of unknown or highly contagious diseases, such as the global pandemic caused by a novel coronavirus, stricter endoscope sterility requirements are imperative [9], underscoring the need for improvements in endoscopic practices and technologies that enhance biosafety to prevent pathogen transmission.

Under the new conditions of modern warfare, GI diseases have become a significant contributor to non-combat casualties and disabilities [10], making the diagnosis and treatment of GI diseases under battlefield conditions increasingly important. Conventional GI endoscopic systems comprise large equipment and are typically confined to specialized endoscopy rooms, limiting their availability, particularly in settings requiring mobility, such as bedside emergencies, medical rescue, military missions in remote or resource-limited areas, and confined spaces such as warships. This can lead to delayed diagnosis and treatment, especially in military actions. Portable endoscopic technologies offer significant advantages in military operations and disaster relief scenarios. In fact, in case of earthquake response or GI bleeding and perforations on the battlefield, such technologies enable timely intervention and precise medical responses, which are crucial for survival [11]. Their portability and minimally invasive design facilitate field deployment, offering diagnostic and therapeutic capabilities when confined to conventional endoscopy units.

To address the challenges of conventional endoscopy, including its large size, limited portability, infection risks, impracticality in bedside emergencies and remote rescue missions, and high maintenance costs [10], we developed a portable endoscopic system named YunSendo. This study evaluates the clinical efficacy and safety of YunSendo in human GI endoscopy.

## Methods

### Patients

This study is an open-label, randomized, self-controlled design, and it is divided into two main sections: a randomized non-inferiority trial and an exploratory study. The first part aims at evaluating the safety and efficacy of the YunSendo system in patients; the second part focuses on assessing the preliminary safety and efficacy of the YunSendo system in critically ill patients.

In the first part of the present study, the patients were selected from the community, with the recruitment period spanning from September to December 2020. A total of 30 patients aged 18–75 years, including 18 male and 12 female patients, participated in the study. After adhering to the inclusion and exclusion criteria, they were grouped based on their intended examination. Of these patients, 15 agreed to undergo both gastroscopy and colonoscopy, while the remaining 15 consented to undergo only gastroscopy. Thirty esophagogastroduodenoscopy (EGD) and 15 colonoscopy procedures were performed, and participants were followed up for 1 week, recording all endoscopy-related adverse reactions. The inclusion criteria were: 1) age 18–75 years; 2) clear evidence of melena, positive fecal occult blood test, hematemesis, or a positive occult blood test in the gastric tube drainage fluid; 3) ongoing bleeding, such as a declining hemoglobin levels, low blood pressure unresponsive to fluid resuscitation, or persistent symptoms of melena/hematemesis; and 4) signed informed consent. Exclusion criteria included absolute contraindications to endoscopy.

For the second section of the present study, the patients were selected from the emergency department, with the recruitment period spanning from January to December 2023. Ten patients with suspected upper GI bleeding were admitted from the emergency department during the global pandemic caused by a novel coronavirus outbreak to simulate a wartime biosecurity scenario. The research flowchart is presented in Additional file 1: Fig. S1.

This study was approved by the Ethics Committee of Chinese PLA General Hospital (S2019-292-02, S2023-007-01). Participants gave informed consent to participate in the study before taking part. This study has been registered in the Chinese Clinical Trial Registry (ChiCTR2000038516, registered September 23, 2020, http://www.chictr.org.cn/showproj.aspx?proj=61752; ChiCTR2300069260, registered March 10, 2023, https://www.chictr.org.cn/showproj.html?proj=190406).

### Portable endoscope system and single-use endoscope

**Fig. 1:**
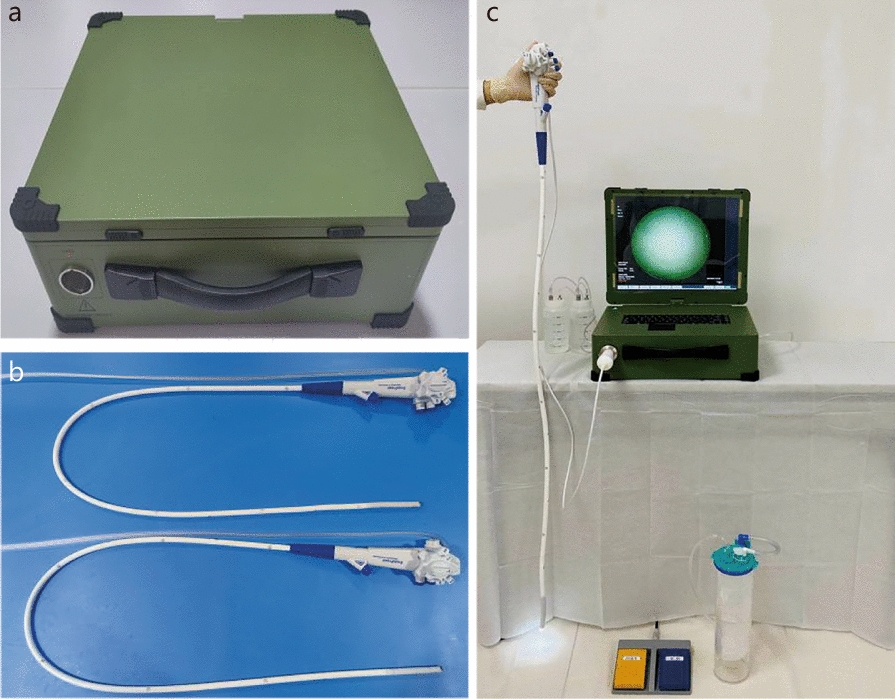
The YunSendo system. **a** Endoscopic host. **b** Gastroscope (above) and colonoscope (below). **c** The assembled YunSendo system

The YunSendo portable endoscope system was developed by the Department of Gastroenterology and Hepatology at the First Medical Center of Chinese PLA General Hospital. The system includes a connective cable, foot pedal, power adapter, power cord, cleansing bottle, suction bottle, and auxiliary water bottle (Fig. 1). The single-use endoscope was manufactured by Huizhou Xzing Technology Co., Ltd., China. It has passed the safety and performance testing by the National Medical Products Administration (NMPA) in China and has obtained a Chinese medical device registration certificate and tested and certified by the European Conformity and the United States Food and Drug Administration. The single-use endoscopes were sterilized using ethylene oxide before packaging and distribution, ensuring readiness for immediate use without the need for additional sterilization or disinfection. The single-use endoscopes are made of biodegradable materials that ensure environmental sustainability. After each procedure, the endoscopes were collected and processed through a stringent recycling protocol. The Yunsendo system offers exceptional portability, supports a direct-current power supply, and features a built-in battery for emergency use. Currently, no similar system is available in the market. Previous studies demonstrated the efficacy and safety of the YunSendo system in animal GI endoscopy [12] and bronchoscopy [13].

The YunSendo system can perform video recording, irrigation, gas insufflation, and suction. With the connective cord linked to the disposable endoscope and activated endoscopy image processing software, the host viewing screen can display real-time images and record videos, capture images, and store them. The host’s length, width, and height were respectively 44 cm, 37.5 cm, and 15 cm (when folded) or 37.5 cm (unfolded). Its weight is 13.8 kg. The system can be powered by alternating currents or built-in batteries, with a continuous working time of approximately 4 h. A detailed comparison of the data between YunSendo and the Olympus endoscope is provided in Additional file 1: Table S1.

### Clinical trial process and staff training

Since the 2 types of endoscopes used in this study are easily distinguishable by appearance, the trial was open-label and not blinded. To minimize inter-patient variability, and in alignment with the clinical trial protocol for the first portable endoscopy device [14], we employed self-controlled study design. Each patient underwent EGD and colonoscopy twice: with the YunSendo system and a single-use endoscope (the experimental group), and the conventional endoscopic Olympus system (the control group); order was randomized by coin toss. Endoscopy was performed under general anesthesia. During endoscopy, images of the GI parts and lesions were captured and stored, and the time for each part was recorded. During these procedures, the operators formulated real-time treatment strategies based on the patient’s condition. These strategies included biopsy, washing, submucosal injection of epinephrine, placement of a feeding tube, and application of hemostatic clips.

Five gastroenterologists, each having performed over 10,000 endoscopic procedures, including various therapeutic endoscopies, conducted all endoscopic procedures. These 5 gastroenterologists underwent training on the YunSendo system and participated in multiple practice sessions during animal experiments to ensure proficiency for human trials. While there was an initial learning curve during the first human trial, the accuracy and safety of the operation were maintained through rigorous training and pre-experiment procedures.

### Operation time

The time taken for each part and for the entire procedure was recorded. Specifically, 1) upper GI parts: the time taken for the endoscope to pass through the oropharynx, esophagus, cardia, gastric body, and pylorus; 2) duodenum: time of entry into the duodenum and subsequent exit; 3) cecal intubation time: the time taken to advance the colonoscope from the anus to the cecum; 4) other parts: time taken to pass through each part; 5) total treatment time: time for emergency endoscopic treatment using the YunSendo system.

### Image quality

Endoscopic images of the upper GI parts, such as the oropharynx; upper, middle, and lower esophagus; cardia; fundus; gastric body (near, middle, and distant parts); gastric angle (anterior, middle, and posterior parts, antral side, body side); gastric antrum; pylorus; and the 4 walls of the first and second duodenal parts were recorded under EGD, amounting to 40 images for each EGD procedure. Eighteen colonoscopy images were collected, including images of the terminal ileum, cecum, appendiceal orifice, ascending colon (upper, middle, and lower), hepatic flexure, transverse colon (left, middle, and right), splenic flexure, descending colon (upper, middle, and lower), sigmoid colon (upper, middle, and lower), and rectum. The image quality was evaluated using sharpness, resolution, and illumination scores. To meet the requirements of the clinical examination, each image was counted as 1 point, and the maximum score was 40 points for EGD and 18 points for colonoscopy.

### Operation performance

Eleven indicators were included to assess the operative performance of both endoscopic systems. These included image-recording time, gas insufflation ability, suction ability, irrigation ability, flexibility of large knobs, flexibility of small knobs, biopsy channels, endoscope flexibility, vision field, bending range of the endoscope, and anti-fog capability of the endoscopic camera. Each item was graded by the operators on a 10-point Likert scale. Nine to ten participants indicated that the operators were very satisfied (9–10); satisfied (7–8); neutral (5–6); dissatisfied (3–4); or very dissatisfied (1–2).

### Detected lesions and safety evaluation

The number of lesions detected was also recorded. Lesions were recorded during the initial endoscopic examination, with treatment administered during the subsequent examination. As the order of the endoscopic procedures was randomized, it would not influence the comparison of lesion detection rates. Adverse reactions, including perforation, bleeding, nausea, vomiting, accidental tracheal injury, and throat injury, were also recorded. Complications would be documented immediately after each procedure to prevent ambiguity in determining which procedure caused the complication. If a complication arises from the first endoscopic procedure, it would be identified following the second procedure and recorded accordingly.

### Clinical outcomes in emergency endoscopic treatment

We focused on the ability of the YunSendo system to perform bedside upper GI endoscopy procedures, including biopsy, endoscopic submucosal injection, hemostatic clip application, and nutritional tube placement. Diagnostic accuracy was assessed based on the identification and grading of bleeding sources using the Forrest or LDRf classification. Clinical outcomes were measured by recording the rates of 1-day, 7-day, and 30-day rebleeding and mortality.

In this study, the primary research indicator was the operation time, while secondary research indicators included image quality, operational performance, lesion detection, safety evaluation, and clinical outcomes in emergency endoscopic treatment. In the design of this study, operation time was selected as the primary evaluation criterion, partly because it directly reflects the efficiency and convenience of endoscopic procedures. Additionally, since the YunSendo system in this study is primarily intended for use in special environments such as battlefields, time efficiency is a key consideration.

Our study involves a new, field endoscope rather than a commercially available or established model. While we have conducted extensive animal studies, safety remains a critical concern in clinical trials. Ensuring diagnostic accuracy is essential for effective treatment. Therefore, we consider safety and diagnostic accuracy as secondary endpoints in this study.

### Sample size calculation

The following procedure was used for EGD and colonoscopy. The sample sizes were determined using PASS 15.0, employing the Non-Inferiority Tests for One Mean method, based on the total operation time of EGD and colonoscopy. Considering clinically acceptable margins, we set non-inferiority margins of 35 s for EGD and 40 s for colonoscopy, with standard deviations of 50 and 40, respectively. We aimed at 90% power for sample size estimation with a one-sided significance level of 0.025. This calculation resulted in required sample sizes of 24 and 13 patients for EGD and colonoscopy, respectively.

The bedside emergency procedures were as follows. As this part of the study is exploratory and focuses on critically ill patients, a smaller sample size of 10 patients was selected. The primary goal for this subgroup was to gather preliminary data and assess the feasibility, effectiveness, and safety of the YunSendo system in emergency settings.

### Statistical analysis

The evaluation parameters of the conventional Olympus and YunSendo groups were recorded and evaluated using either objective measurements or third-party assessments. SPSS software (version 26.0) was used for the statistical analysis. Normally distributed continuous variables were presented as mean ± standard deviation (SD), and nonnormally distributed data were presented as median (IQR). Non-inferiority of the total operation time, image quality, and operation performance between the Olympus and YunSendo groups was assessed using a one-sided paired *t*-test or the Wilcoxon signed-rank test, with non-inferiority margins set at 35.0 and 40.0 for the total operation time of EGD and colonoscopy, respectively, 1.0 for image quality, and 1.5 for operation performance. Statistical significance was set at *P* < 0.05.

## Results

The EGD group consisted of 18 procedures for male and 12 procedures forfemale patients, while the colonoscopy group had 11 procedures for male and 4 procedures for female patients. The average age of these patients assigned to the EGD group was (32.93 ± 6.23) years, while the average age of those assigned to the colonoscopy group was (32.27 ± 6.38) years, with no significant statistical difference between the 2 groups (*P* > 0.05). A total of 7 male patients and 3 female patients participated. The majority of the patients were aged between 50 and 72 years.

### Operation time

Five GI endoscopy experts were involved in the initial utilization of the YunSendo endoscope. Consequently, the operation times of 3 patients were excluded from the analysis. Moreover, 1 participant who underwent colonoscopy was evaluated only for cecal intubation time and total operation time, which precluded assessment of the remaining colonic parts.

The total operation time and segment-specific time for EGD and colonoscopy in the conventional Olympus and YunSendo groups were recorded (Tables 1 and 2). The total operation time of EGD with Olympus was (327.19 ± 82.97) s, while that of EGD with YunSendo-GI was (346.04 ± 90.98) s. The total operation time of colonoscopy with Olympus was (509.93 ± 185.08) s, while that of EGD with YunSendo-GI was (585.79 ± 165.14) s. The non-inferiority test for the total operation time yielded a *P*-value of 0.154 for EGD and 0.777 for colonoscopy. These results indicated that the YunSendo endoscopic system required longer operation times than the conventional endoscopic system for both EGD and colonoscopy.

**Table 1:** Comparison of EGD operation time between the YunSendo-GI group and the Olympus group (s, *n* = 27)

Group	Passed-through part						
	Oropharynx [median (IQR)]	Esophagus [median (IQR)]	Cardia [median (IQR)]	Gastric body [median (IQR)]	Pylorus [median (IQR)]	Duodenum [median (IQR)]	Total operation time (mean ± SD)
EGD with Olympus	28.00	50.00	2.00	33.00	2.00	53.00	327.19 ± 82.97
	(22.50–34.00)	(40.50–60.50)	(2.00–4.00)	(23.00–48.00)	(2.00–5.50)	(30.50–66.50)	
EGD with YunSendo-GI	30.00	45.00	2.00	39.00	5.00	52.00	346.04 ± 90.98
	(25.00–42.50)	(32.50–66.00)	(2.00–3.00)	(29.00–46.50)	(2.50–8.50)	(44.00–83.50)	
*P-*value for non-inferiority							0.154^*^

^*^Non-inferiority margin: 35.0 s

*GI* gastrointestinal, *EGD* esophagogastroduodenoscopy

**Table 2:** Comparison of colonoscopy operation time between the YunSendo-GI group and the Olympus group (s, *n* = 14)

Group	Passed-through part								
	Rectum [median (IQR)]	Sigmoid colon [median (IQR)]	Descending colon (mean ± SD)	Splenic flexure [median (IQR)]	Transverse colon [median (IQR)]	Hepatic flexure [median (IQR)]	Ascending colon [median (IQR)]	Cecal intubation time [median (IQR)]	Total operation time (mean ± SD)
Colonoscopy with Olympus	15.00	61.50	37.14 ± 31.43	6.00	29.00	6.50	14.00	148.50	509.93 ± 185.08
	(11.25–25.75)	(31.25–82.75)		(5.25–9.50)	(12.00–96.50)	(4.00–13.50)	(6.75–17.00)	(119.50–268.00)	
Colonoscopy with YunSendo-GI	16.50	68.50	41.07 ± 24.84	11.00	46.50	11.00	9.50	213.50	585.79 ± 165.14
	(11.75–23.75)	(58.00–84.75)		(5.00–17.75)	(22.00–72.75)	(7.00–19.00)	(4.00–24.50)	(193.50–311.75)	
*P*-value for non-inferiority									0.777^*^

^*^Non-inferiority margin: 35.0 s for EGD and 40.0 s for colonoscopy

*GI* gastrointestinal

For bedside emergencies, the YunSendo endoscopic system facilitated the successful diagnosis and treatment of 10 critically ill patients with upper GI hemorrhage. Additional file 1: Table S2 presents detailed operation times.

### Image quality

Additional file 1: Fig. S2 shows EGD and colonoscopic images from both groups. One participant was excluded because of inadequate bowel preparation for colonoscopy, which affected image assessment. In both upper GI and intestinal examinations, the Olympus endoscope and the YunSendo system had median scores of 40 and 18, respectively, for image sharpness, image resolution, and image illumination, with non-inferiority test results all being less than 0.05 (Tables 3 and 4). These results indicate that the image sharpness, resolution, and illumination of the YunSendo system are noninferior to those of the conventional Olympus system.

**Table 3:** Image quality scores and operation performance scores of the YunSendo-GI group and the Olympus group [median (IQR)]

Group	Image sharpness (EGD/Colonoscopy)	Image resolution (EGD/Colonoscopy)	Image illumination (EGD/Colonoscopy)
Olympus endoscope	40 (39–40)/18 (17–18)	40 (40–40)/18 (18–18)	40 (39–40)/18 (17–18)
YunSendo-GI endoscope	40 (39–40)/18 (18–18)	40 (39.5–40)/18 (18–18)	40 (40–40)/18 (18–18)
*P*-value for non-inferiority^*^	0.0001/0.0013	0.0001/0.0006	< 0.0001/0.0003

^*^Non-inferiority margin: 1

*GI* gastrointestinal, *EGD* esophagogastroduodenoscopy

**Table 4:** Operation performance scores of the Olympus group and the YunSendo-GI group [median (IQR)]

Group	Image-recording time	Gas insufflation ability	Suction ability	Irrigation ability	Flexibility of large knob	Flexibility of small knob	Biopsy channel	Flexibility	Vision field	Bending range	Anti fog capability
Olympus enscope	10 (9–10)	9 (9–9)	9 (9–9)	9 (9–9)	9 (9–9)	9 (9–10)	10 (10–10)	9 (9–10)	9 (9–9)	9 (9–9)	9 (9–9)
YunSendo-GI endoscope	9 (9–9)	9 (9–9)	9 (9–9)	9 (9–9)	9 (9–9)	9 (9–9)	10 (9–10)	9 (8–9)	9 (9–10)	9 (9–10)	8 (7–8)
*P-*value	0.027	0.005	0.023	0.024	0.024	0.027	0.024	0.024	0.027	0.027	0.383

*GI* gastrointestinal

### Operative performance

The *P*-values for the operative performance scores of image-recording time, gas insufflation ability, suction ability, irrigation ability, the flexibility of large knobs, the flexibility of small knobs, biopsy channel, flexibility, vision field, and endoscope bending range between the YunSendo group and the conventional Olympus group were 0.027, 0.005, 0.023, 0.024, 0.024, 0.027, 0.024, 0.024, 0.027, and 0.027, respectively. This indicates that the YunSendo group was not significantly inferior to the conventional endoscope group in these aspects (*P* < 0.05). However, regarding the anti-fog capability of the endoscopic camera, the YunSendo system was inferior to the conventional Olympus system (Table 5).

**Table 5:** Operation performance scores of the Olympus group and the YunSendo-GI group

Group	Image-recording time [median (IQR)]	Gas insufflation ability [median (IQR)]	Suction ability [median (IQR)]	Irrigation ability [median (IQR)]	Flexibility of large knobs [median (IQR)]	Flexibility of small knobs [median (IQR)]	Biopsy channel [median (IQR)]	Flexibility [median (IQR)]	Vision field [median (IQR)]	Bending range (mean ± SD)	Anti-fog capability [median (IQR)]
Olympus endoscope	10 (9–10)	9 (9–9)	9 (9–9)	9 (9–9)	9 (9–9)	9 (9–10)	10 (10–10)	9 (9–10)	9 (9–9)	9.20 ± 0.84	9 (9–9)
YunSendo-GI endoscope	9 (9–9)	9 (9–9)	9 (9–9)	9 (9–9)	9 (9–9)	9 (9–9)	10 (9–10)	9 (8–9)	9 (9–10)	9.00 ± 0.70	8 (7–8)
*P*-value	0.027	0.005	0.023	0.024	0.024	0.027	0.024	0.024	0.027	0.027	0.383

*GI* gastrointestinal

### Detected lesions and safety evaluation

**Fig. 2:**
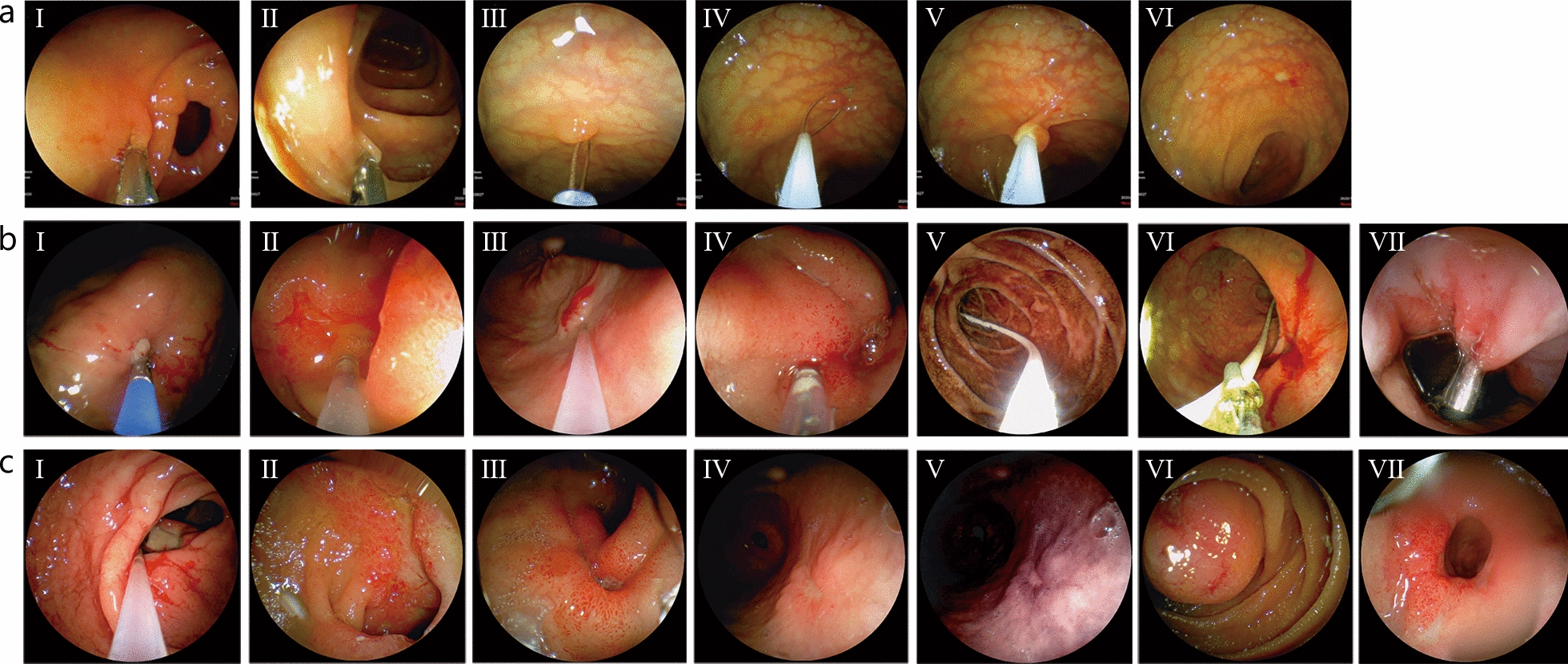
Procedures with YunSendo. **aI** Biopsy using EGD with YunSendo. **aII** Polyp removal using colonoscopy with YunSendo. **aIII**–**VI** Polypectomy performed using YunSendo Emergency procedure with YunSendo and representative lesions. **bI** Biopsy procedure. **bII**–**IV** Application of submucosal injections in patients. **bV**–**VI** Placement of jejunal feeding tubes in patients. **bVII** Application of a hemostatic clip to treat bleeding sites. **cI**–**III** Duodenal ulcers in various patients, gastric ulcer in the same patient under white light (**cIV**) and SWI (**cV**) modes. **cVI** Duodenal mass in a patient. **cVII** Pyloric ulcer in a patient. EGD esophagogastroduodenoscopy, SWI single white image

The conventional Olympus and YunSendo groups detected lesions equally well during EGD (Additional file 1: Table S3). Figure 2aI shows a biopsy performed during EGD using the YunSendo system. EGD performed with the Olympus system served as the reference standard. The diagnostic sensitivity of EGD using the YunSendo system was 100%, confirming the diagnostic value of the YunSendo system. No statistically significant difference was observed in the number of lesions (polyps) detected during colonoscopy between the Olympus and YunSendo groups (*P* = 0.0276), confirming the colonoscopic diagnostic accuracy of the YunSendo system. All detected polyps were removed using the YunSendo (Fig. 2aII and VI) or the conventional Olympus endoscopic system. Additional file 1: Table S4 presents the number, size in diameter, and location of polyps in two groups. No adverse reactions or complications occurred during the procedures.

All participants were followed up 1 week after the examination, and no endoscopy-related adverse reactions were reported. For each patient, comprehensive documentation of adverse events and follow-up assessments was conducted to ensure that any potential adverse reactions related to the YunSendo endoscope were thoroughly monitored and recorded. The diagnostic sensitivity of the YunSendo system was also evaluated, and the results were comparable to those of the Olympus endoscope, providing strong support for patient treatment. And diagnostic accuracy was also included as a co-primary outcome of the study.

### Application of YunSendo in emergency critically ill patients

Among the 10 emergency patients, 6 underwent endoscopic treatment, 4 underwent biopsies, 5 underwent submucosal injections, and 2 had jejunal feeding tubes placed. One patient received a hemostatic clip. Owing to varying patient conditions and differing tolerances to endoscopic procedures, the duration of these treatments varied significantly (Table 6; Fig. 2b).

**Table 6:** Details of the patients undergoing treatment

Patient ID	Treatment time (s)	Biopsy	No. of biopsie	Time	Submucosal injection	Time (s)	Feeding tube	Time (s)	Hemostatic clip	Time (s)
1	479	No	NA	NA	Yes	297	Yes	145	Yes	37
2	71	No	NA	NA	Yes	71	No	NA	No	NA
3	191	Yes	1	21	Yes	191	No	NA	No	NA
7	743	Yes	2	51	No	NA	Yes	692	No	NA
8	369	Yes	1	23	Yes	346	No	NA	No	NA
9	116	Yes	3	60	Yes	56	No	NA	No	NA

*NA* not available

Among the 10 patients, 8 were diagnosed with peptic ulcers, and Fig. 2c shows images of lesions from typical patients. All patients underwent Forrest classification, as shown in Table 7. The specific conditions were as follows: 3 patients experienced bleeding from duodenal ulcers, 4 from gastric ulcers, and 1 from both gastric and duodenal ulcers. The remaining 2 patients presented unique cases. One had a duodenal mass with a central umbilical depression and ulcer, necessitating surgical evaluation, and the other showed no significant ulcers or bleeding on portable endoscopy. The latter had previously undergone endoscopic submucosal dissection for a gastric antral mass, which raised concerns about potential surgical site bleeding. However, upon reexamination using the YunSendo gastroscope, we confirmed that the tissue clips remained securely in place, effectively sealing the wound, and no obvious source of bleeding was observed. And, further examination into the duodenum was not performed. The patient’s condition was monitored, and a subsequent endoscopy was scheduled. After 7 d, the patient exhibited no further bleeding and was discharged. A 30-day follow-up revealed no episodes of bleeding or melena.

**Table 7:** Causes of bleeding and Forrest grading

Patient ID	Causes of bleeding	Forrest grading
Grading		
1	Duodenal ulcer	IIb
2	Duodenal ulcer	IIc
3	Gastric antrum-body junction ulcer	III
4	Gastric lesser curvature ulcer	IIa
5	Duodenal mass	NA
6	No obvious abnormalities	NA
7	Gastric ulcer	IIc
8	Duodenal bulb ulcer	III
9	Gastric ulcer; duodenal ulcer	III

*NA* not available

After endoscopic treatment with the YunSendo system, the 1-day, 7-day, and 30-day mortality rates were all zero, and there were no instances of rebleeding at 1-day or 30-day follow-up, demonstrating the system’s effectiveness in both emergency and long-term hemostasis. On the 7th day following endoscopic treatment, only 1 patient exhibited suspected rebleeding, which was identified by the presence of melena on the 3rd day post-treatment. Bleeding was attributed to oozing from a duodenal bulb ulcer or incomplete evacuation of intestinal blood [15]. The patient received a submucosal injection and hemostatic clips during endoscopy with no significant decrease in hemoglobin levels. Consequently, hemostatic medication was administered, and the patient was observed. No further episodes of melena or hematochezia occurred.

These 10 critically ill patients were admitted during the period following the easing of epidemic control measures in China (early 2023), a period preceding herd immunity, when community transmission of the novel coronavirus and infection rates among the population remained relatively high. None of the 10 patients had been infected with the novel coronavirus before their surgeries, and none developed postoperative infections.

## Discussion

The core principle of the current study is based on portable endoscopy technology, which provides efficient diagnostic and therapeutic solutions without the limitations of traditional endoscopic equipment. This study compares the YunSendo system with traditional endoscopy systems in terms of operation time, image quality, operation performance, detected lesions, and safety evaluation through a randomized, controlled, non-inferiority comparison. Additionally, the YunSendo system was utilized for bedside emergency hemostatic treatment of upper GI bleeding to evaluate its effectiveness and safety in this critical application.

Compared to traditional endoscopy, our novel portable endoscope system is smaller and more convenient, making it suitable for rapid intervention in emergencies and special environments while providing efficient and safe diagnostic and therapeutic solutions.The YunSendo endoscopy system is nearly on par with conventional endoscopes in terms of operation time, image quality, operation performance, detected lesions, and safety evaluation, and can diagnose and treat critically ill patients. Based on the research results, it is foreseeable that portable endoscopy devices are poised to thrive in special environments and on the battlefield, offering rapid and accurate diagnosis and treatment during emergencies and in post-disaster and telemedicine settings, potentially becoming an indispensable tool in global healthcare systems.

The comparison of the parameters between this portable endoscope and the Olympus endoscope revealed that, although the field of view of the portable endoscope was slightly smaller, its larger adjustable bending range provided greater flexibility, effectively compensating for the difference in the field of view. Additionally, the portable endoscope featured a broader depth of field, enabling the observation of GI mucosa at closer distances, which is advantageous for lesion detection. Furthermore, this portable endoscope was not only compact and lightweight but also integrated irrigation, gas insufflation, and suction functions, all of which met or exceeded standard usage requirements.

Our findings showed that the YunSendo system took longer to perform EGD and colonoscopy. However, the YunSendo system did not exceed the standard duration typically required for conventional gastroscopy and colonoscopy procedures, which is 6–10 min for gastroscopy [16] and 15–30 min for colonoscopy [17]. Although the operators had already mastered the use of the new YunSendo system through extensive animal testing [12], this was the first time the procedures were performed on human subjects. This introduces a learning curve as there are differences between animal models and human participants. Notably, the learning curve of endoscopists varies significantly [18–20]. Achieving proficiency in standard upper GI endoscopy typically requires the completion of at least 200 procedures [21]. However, performing advanced endoscopic procedures requires further training [22]. As the number of procedures performed with the YunSendo system increases, operators will become more proficient, reducing the time required for EGD and colonoscopy.

The newly developed endoscopic YunSendo system can perform EGD and colonoscopy effectively and safely. Although the YunSendo system is slightly inferior to conventional endoscopy in terms of the anti-fog capability, the overall operation and image quality remain unaffected. Moreover, lesion-detection ability is comparable to that of conventional endoscopes and is sufficient to meet the needs of clinical GI endoscopy. In this clinical experiment, there were no endoscopy-induced adverse reactions or complications such as perforation, bleeding, nausea, vomiting, accidental tracheal, or laryngopharyngeal injury.

Xu et al.’s [11] study on the portable endoscope conducted Forrest grade I hemostasis tests on pigs, obtaining good results. However, an insufficient number of animal experiments were performed, and the peptic ulcer bleeding treated was classified as grade I risk, making it impossible to predict its hemostatic capability under higher-grade bleeding risks. In contrast, our study not only treated critically ill patients but also included lesions with Forrest grades IIa, IIb, IIc, and III, demonstrating that the YunSendo system can successfully diagnose and treat acute upper GI bleeding. The endoscopic diagnosis and treatment procedures were completed, the causes of bleeding were determined, and images were recorded. The Forrest classification was used for patients with lesions. Emergency operations such as biopsy, submucosal injection, hemostatic clip placement, and nutritional tube placement were performed. These results indicate that the YunSendo system can manage patients with acute nonvariceal upper GI bleeding.

Compared to the conventional endoscope system, the YunSendo system is portable and compatible. Zou et al. [23] reported a portable endoscopy system weighing approximately 35 kg, with an overall size of 68 cm × 42 cm × 32 cm. In comparison, our YunSendo system weighs only 13.8 kg and is more compact (44 cm × 37.5 cm × 15 cm). The YunSendo system host is durable, easy to maintain, and compact, making it ideal for storage purposes. In addition, host unit was designed for versatile applications. Currently, the system is compatible with both single-use GI endoscopes and the single-use bronchoscopes developed by our group [13]. This makes it convenient and capable of performing endoscopy under various conditions, such as bedside emergencies, remote areas, isolation rooms, and other settings where conventional endoscopy equipment is unavailable.

Despite the complexity and strictness of conventional reprocessing procedures, the comprehensive infection rate in GI endoscopy remains approximately 0.2% [24]. Procedures such as endoscopic retrograde cholangiopancreatography (ERCP) exhibit higher infection rates (0.8%), whereas non-ERCP upper and lower GI endoscopies have infection rates of 0.123% and 0.073%, respectively [24]. Outbreaks linked to organisms such as *Klebsiella pneumoniae* and *Pseudomonas aeruginosa* have been documented globally and are often attributed to reprocessing lapses and design issues with the endoscopes themselves [25]. Reprocessing is challenging because of variable failure rates, persistent contamination from biofilm formation, endoscopic defects, and inadequate drying processes [25]. Although high-level disinfection of GI endoscopes results in relatively low infection rates, it does not meet sterilization standards, highlighting the potential of single-use endoscopes as a promising and viable solution. Furthermore, the cleaning and maintenance of reusable conventional endoscopes are labor-intensive and costly [26]. Each endoscopic procedure requires a rigorous and time-consuming cleaning process to prevent cross-contamination and infection [27]. This involves large quantities of chemical disinfectants and wastewater, specialized equipment, extensive manual labor, and stringent adherence to disinfection protocols [28].

The global pandemic caused by a novel coronavirus represented a severe public health event in world history, with people worldwide affected by symptoms of varying degrees, primarily manifesting as fever, cough, fatigue, and difficulty breathing, among others [29]. Additionally, a potential for GI transmission exists, making GI endoscopy procedures more prone to causing infections among healthcare workers and cross-contamination between patients [30]. Throughout our research, no infections were reported by the healthcare personnel involved, and all participating patients remained uninfected, representing the advantages of using single-use endoscopes.

Several advancements in portable and disposable endoscopic technologies have been made by research teams at other institutions over the past decade. Kang et al. [31] evaluated the feasibility of a portable and disposable ultrathin endoscope (DE) system for diagnosing upper GI diseases. However, the endoscope did not have the functions of irrigation, gas insufflation, suction, endoscopic biopsy, or treatment. DE can accurately diagnose esophageal lesions only but cannot precisely detect gastric and duodenal lesions [31]. Another study [32] showed that a disposable EGD endoscopy system completed surgical and interventional procedures such as hemostasis, foreign body retrieval, gastroenteric tube placement, and percutaneous endoscopic gastrostomy without requiring traditional upper GI endoscopes. However, the need for an additional external display screen complicates the overall design. Zou et al. [23] developed a portable endoscopy system with both diagnostic and therapeutic functions; however, it is designed solely for upper GI examinations, whereas our system is capable of performing both upper and lower GI procedures. Another study compared single-use and reusable duodenoscopes in ERCP and concluded that single-use duodenoscopes are feasible for low-complexity ERCP procedures, albeit with limitations in their performance characteristics [33]. Han et al. [32] studied a disposable EGD system, which is capable of diagnosing and treating upper GI lesions. It is highly portable and designed to eliminate the risk of cross-infection caused by reusable EGD. However, it is also not compatible with colonoscopy, and there is no comparative study with traditional endoscopes. Additionally, its design is not as suitable for extreme environments such as battlefields and disasters as the YunSendo system. The comparison of other characteristics can be found in Additional file 1: Table S5.

The present study’s authors believe that, in the future, endoscopic systems will undoubtedly evolve towards portability, high integration, and high artificial intelligence, with a single host capable of being compatible with various endoscopic devices (such as gastroscopes, colonoscopes, bronchoscopes, cystoscopes, laparoscopes, etc.). Against this background, the functions of these systems will gradually become more comprehensive, enabling them to perform a variety of complex therapeutic procedures. Further, with the development of artificial intelligence, large AI models will be integrated into portable endoscopy systems to assist operators in decision-making.

The current study provides the following insights and implications: first, our research fills the gap concerning the fact that no commercially available endoscopic system is specifically designed for use in the battlefield, disaster relief, or unique environments with biosafety concerns. Second, the present research process proves to have withstood the test of the pandemic, with no patients being infected, demonstrating its ability to address biosafety concerns. Third, the YunSendo system is compatible with gastroscopy, colonoscopy, and bronchoscopy [12, 13], and we are researching its compatibility with more types of endoscopes or cannulas. Since the endoscope is single-use, there is no need to carry dedicated cleaning and disinfection equipment or consider disinfection or cross-contamination issues. Fourth, due to its characteristics and leading clinical translation progress, this endoscopy system is particularly suited for medical treatment in unconventional conditions, such as battlefield care and natural disasters.

The study has the following limitations: first, there is an inherent inability to use a blinded design because of its nature as a self-controlled study. The gastroenterologists knew the type of endoscope used in each procedure, which could have introduced bias. Second, the procedures were performed by highly experienced gastroenterologists, which may limit the generalizability of the results to all endoscopists. Less experienced practitioners may achieve different outcomes using the same endoscopic system. Third, the therapeutic procedures used in this study were limited to polyp removal, placement of jejunal feeding tubes, and endoscopic hemostasis. More complex endoscopic interventions require further investigations to fully assess the safety and efficacy of the YunSendo system for a broader range of therapeutic applications. The necessity for further validation arises in both rescue and military scenarios. Additionally, the endoscopic tube is made of disposable materials, and the YunSendo system has inferior anti-fog performance compared to Olympus endoscopes. Further improvements in coating materials will be needed in the future.

## Conclusions

This study demonstrated that the YunSendo system performs comparably to the Olympus system in key metrics, highlighting its utility in urgent clinical scenarios and its capability for biosecurity prevention. Thus, the YunSendo system is a viable alternative for both standard endoscopic procedures and emergency interventions, particularly in battlefield and biosafety scenarios.

## Supplementary Information

**Additional file 1.** **Table S1** Comparison of Parameters Between Portable Endoscopes and Olympus Endoscopes. patientpatient. **Table S2** Therapeutic operation time using the YunSendo-GI system (s, *n* = 10). **Table S3** EGD detected diseases with Olympus and YunSendo-GI systems. **Table S4** The number, size in diameter, and location of polyps under colonoscopy with Olympus and YunSendo-GI. **Table S5** The characteristics of portable gastrointestinal endoscopes in the past decade. **Fig. S1** Research flowchart.** Fig. S2 **Images from YunSendo-GI and Olympus.

## Data Availability

The datasets used and/or analysed during the current study are available from the corresponding author on reasonable request.

## Abbreviations

DE: Disposable ultrathin endoscope
EGD: Esophagogastroduodenoscopy
ERCP: Endoscopic retrograde cholangiopancreatography
GI: Gastrointestinal
