# Supplementary material for: Evaluation and application of an innovative portable field endoscope: addressing battlefield and biosafety concerns
Source: Mil Med Res. 2025 Sep 15;12:57. doi: 10.1186/s40779-025-00644-w (PMC12434913; doi:10.1186/s40779-025-00644-w)
Supplement: Supplementary file 1 — Additional file 1. Table S1 Comparison of Parameters Between Portable Endoscopes and Olympus Endoscopes. patientpatient. Table S2 Therapeutic operation time using the YunSendo-GI system (s, n = 10). Table S3 EGD detected diseases with Olympus and YunSendo-GI systems. Table S4 The number, size in diameter, and location of polyps under colonoscopy with Olympus and YunSendo-GI. Table S5 The characteristics of portable gastrointestinal endoscopes in the past decade. Fig. S1 Research flowchart. Fig. S2 Images from YunSendo-GI and Olympus. [file 40779_2025_644_MOESM1_ESM.pdf]

Table S1 Comparison of parameters between portable endoscopes and Olympus endoscopes

Parameter	Portable gastroscope	GIF-H290*	Portable colonoscope	CF-H290I*
Optical system				
Field of view (°)	120	140	120	140
Direction	Forward	Forward	Forward	Forward
Depth of field (mm)	3 – 100	7 – 100	3 – 100	7 – 100
Insertion section				
Shaft diameter (mm)	10.9	9.7	12.9	12.8
Working length (mm)	1300	1030	1325	1330
Bending section				
Bending angle (°)	Up: 195<br>Down: 190	Up: 210<br>Down: 90	Up: 195<br>Down: 190	Up: 180<br>Down: 180
Bending angle (°)	Left: 180<br>Right: 180	Left: 100<br>Right: 100	Left: 170<br>Right: 170	Left: 160<br>Right: 160
Instrument channel				
Working channel diameter (mm)	3	2.2	3.2	3.2
Minimum visible distance (mm)	3	4	3	4

*The data for the Olympus endoscopes is sourced from https://www.tenmed.net/

Table S2 Therapeutic operation time using the YunSendo-GI system (s, *n* = 10)

Passed-through part	Oropharynx (mean ± SD)	Esophagus [median (IQR)]	Stomach [median (IQR)]	Pylorus (mean ± SD)	Duodenum [median (IQR)]	Total treatment time [median (IQR)]	Total operation time (mean ± SD)
Operation time	26.5 ± 22.31	29.50 (19.00, 37.00)	47.50 (24, 160.00)	19.90 ± 15.00	35.00 (27.00, 77.00)	328.17 ± 233.26	670 (616.75, 993.00)

GI gastrointestinal

Table S3 EGD detected diseases with Olympus and YunSendo-GI systems

**Lesions**	**Olympus endoscope**	**YunSendo-GI endoscope**	**Sensitivity (%)**
Ectopic gastric mucosa in the upper esophagus	7	7	100
Reflux esophagitis	1	1	100
Chronic gastritis	23	23	100
Acute gastritis	2	2	100
Erosive gastritis	5	5	100
Gastric polyps	4	4	100
Bile reflux	2	2	100
Duodenal polyps	1	1	100
Duodenitis	4	4	100
Duodenal ulcer	1	1	100

EGD esophagogastroduodenoscopy, *GI* gastrointestinal

Table S4 The number, size in diameter, and location of polyps under colonoscopy with Olympus and YunSendo-GI

**Location**	**Olympus**		**YunSendo-GI**	
	n	**Size in diameter (cm)**	***n***	**Size in diameter (cm)**
Ascending colon	0		2	0.3, 0.4
Hepatic flexure	2	0.3, 0.3	2	0.3, 0.3
Transverse colon	1	0.4	0	
Sigmoid colon	2	0.2, 0.3	3	0.3, 0.3, 0.6
Rectum	0		1	0.3

GI gastrointestinal

Table S5 The characteristics of portable gastrointestinal endoscopes in the past decade

Name	Country	Publication year	Advantage	Disadvantage	References
A bedside portable endoscopy device	Korea	2014	Effectively diagnoses esophageal lesions;<br>Highly portable	Low integration;<br>Limited diagnostic ability for upper gastrointestinal (stomach and duodenum) lesions;<br>Only capable of upper gastrointestinal examination;<br>Cannot perform therapeutic procedures	[1]
An operable, portable, and disposable ultrathin endoscope	Korea	2019	This is an improved version of the device described in the article with PMID: 25009396, offering more flexible operation and stronger diagnostic capabilities	Low integration;<br>Only capable of upper gastrointestinal examination;<br>Cannot perform therapeutic procedures	[2]
A single-use and reusable duodenoscope	USA	2021	Innovatively proposed as a single-use duodenoscope	Requires experienced hands;<br>Can only perform low-complexity ERCP procedures	[3]
A disposable esophagogastroduodenoscopy system	China	2023	High integration;<br>Can meet both diagnostic and therapeutic needs simultaneously	Host and display are separate;<br>Requires an endoscope trolley;<br>Not suitable for use outside hospitals	[4]
A portable upper gastrointestinal endoscopy system	China	2023	High integration;<br>Highly portable	Only capable of upper gastrointestinal examination;<br>Cannot perform therapeutic procedures	[5]
A portable field endoscope	China	NA	High integration;<br>Highly portable;<br>High compatibility	Clinical translation progress needs further advancement	Our research

ERCP endoscopic retrograde cholangiopancreatography, *NA* not available

References

1. Comparison of a novel bedside portable endoscopy device with nasogastric aspiration for identifying upper gastrointestinal bleeding. World J Gastroenterol. 2014;20(25):8221-8.
2. An operable, portable, and disposable ultrathin endoscope for evaluation of the upper gastrointestinal tract. Dig Dis Sci. 2019;64(7):1901-7.
3. Equivalent performance of single-use and reusable duodenoscopes in a randomized trial. Gut. 2021;70(5):838-44.
4. Evaluation of a novel disposable esophagogastroduodenoscopy system in emergency, bedside, and intraoperative settings: pilot study (with videos). Dig Endosc. 2023;35(7):857-65.
5. Gastrointestinal endoscopy-associated infections: update on an emerging issue. Dig Dis Sci. 2022;67(5):1718-32.

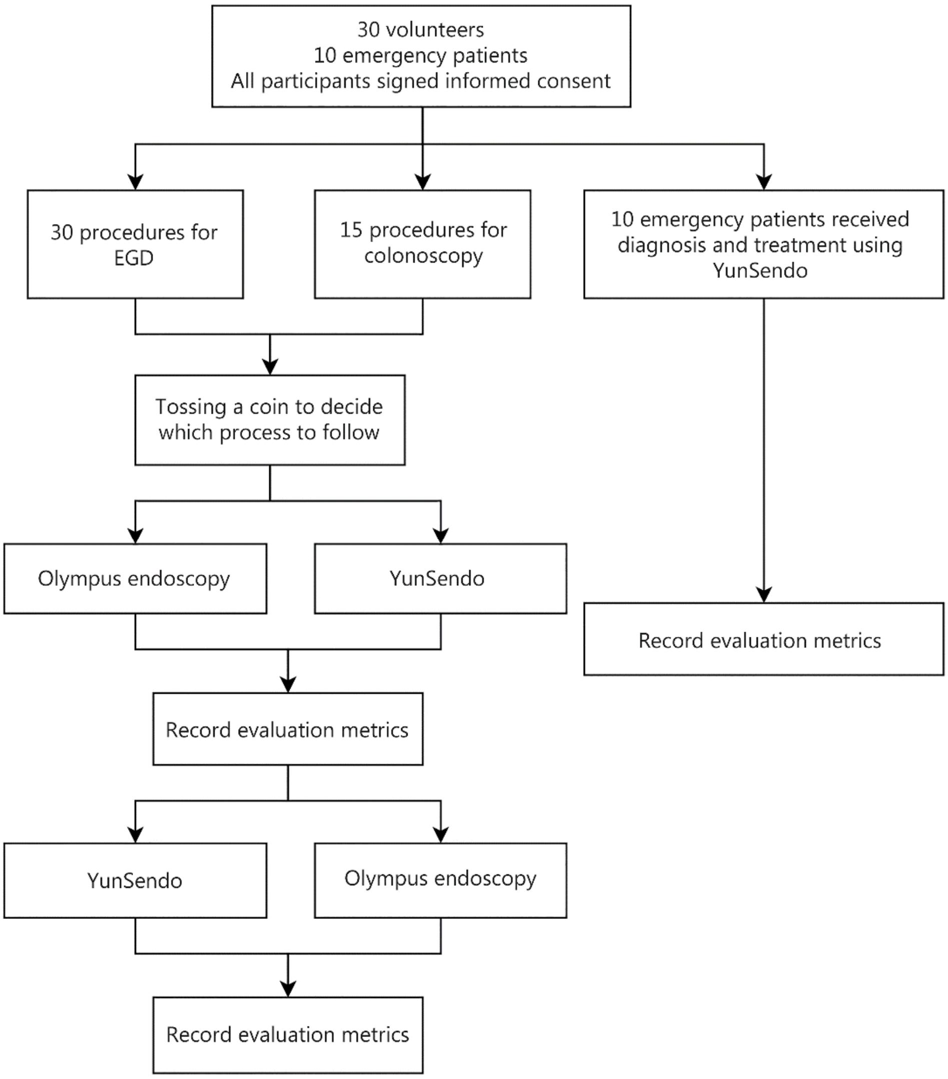

Fig. S1 Research flowchart. EGD esophagogastroduodenoscopy

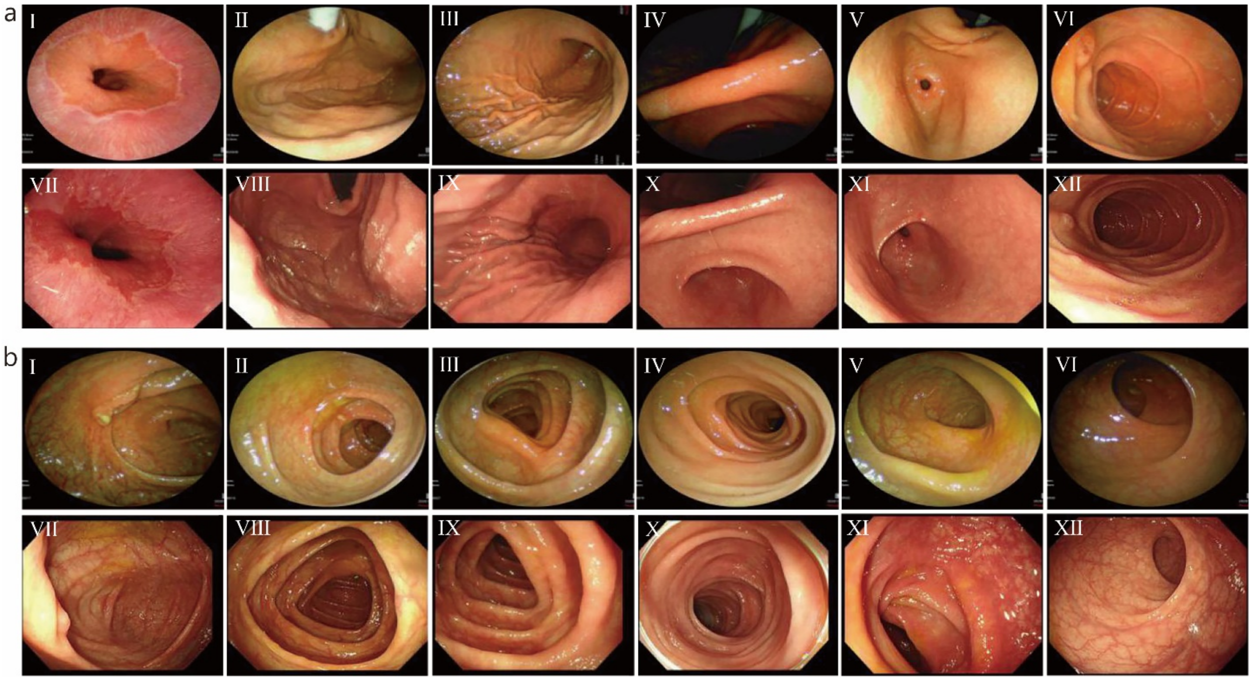

Fig. S2 Images from YunSendo and Olympus. **a** Images from YunSendo EGD (I – VI) and Olympus EGD (VII – XII). **b** Images from colonoscopy using YunSendo (I – VI) and Olympus (VII – XII). EGD esophagogastroduodenoscopy
